# Cinacalcet Treatment of Primary Hyperparathyroidism

**DOI:** 10.1155/2011/415719

**Published:** 2011-03-06

**Authors:** H. M. Rothe, O. Liangos, P. Biggar, A. Petermann, M. Ketteler

**Affiliations:** Division of Nephrology and Hypertension, Medical Department, Klinikum Coburg III, D-96450 Coburg, Germany

## Abstract

Although parathyroidectomy remains the only curative approach to most primary hyperparathyroidism cases, medical treatment with cinacalcet HCl has been proven to be a reasonable alternative for several patient subgroups. Cinacalcet almost always controls hypercalcemia and hypophosphatemia sufficiently. PTH levels are lowered, and cognitive parameters improve. While an increase in bone mineral density DEXA scan scores was not demonstrated in cinacalcet trials, the same applies to more than half of patients after parathyroidectomy. Medical therapy should be first choice in patients with hyperplasia in all glands rather than an isolated adenoma (10–15%), patients with persisting HPT following unsuccessful surgery or inoperable cases due to comorbidities, and patients detected in lab screens for hypercalcemia before developing symptoms who should be treated early but are usually reluctant to undergo surgery. Nephrolithiasis was not found to occur more frequently in cinacalcet trial groups, but urine calcium excretion as one major risk factor of this complication of primary HPT may increase on cinacalcet. Patients carrying the rs1042636 polymorphism of the calcium-sensing receptor gene respond more sensitively to cinacalcet and have a higher risk of calcium stone formation. Cinacalcet is usually administered twice daily but three or four doses per day should be discussed to mimic the beneficial pulsatile PTH-pattern.

## 1. Introduction

Since the approval of cinacalcet HCl for the therapy of secondary hyperparathyroidism (sHPT) and hypercalcemia due to parathyroid carcinoma in 2004, calcimimetic therapy has become daily routine especially for clinical nephrologists worldwide. While pHPT was included as an indication by the European Drug Agency in June 2008, this has not been the case so far in the US. The drug is generally well tolerated, and, although there is still considerable inter-individual variation in dose response ratio, this is much less pronounced as compared with the earlier compound R-568, which remained experimental and was not introduced into clinical practice for that reason. PTH target ranges can be achieved in about 70% of secondary HPT patients, adverse events include mainly nausea, diarrhea, and rarely paresthesias in this patient population.

 From the beginning of the development of this new class of drugs, calcimimetics have been considered for the treatment not only of secondary but also of primary HPT. As early as 1997 Silverberg et al. reported a short-term inhibition of PTH secretion by R-568 in 20 postmenopausal women [1]. The first evidence of a long-term benefit from activated calcium-sensing receptor status in primary HPT patients was provided by a genetic study: Yamauchi et al. found lower PTH and serum calcium levels in 105 Japanese pHPT patients with the calcium-sensing receptor polymorphism Arg990Gly in 2001, as compared with patients without the polymorphism [2]. This polymorphism, which leads to an exchange of arginine by glycine in position 990 of the receptor molecule, results in a permanent allosteric activation of the receptor. Conformational change of the receptor molecule leads to an increased sensitivity to calcium ions, which can be further increased due to the transient effect of a calcimimetic compound binding to the receptor.

One might speculate, that administering a CaSR activating agent in a state of reduced CaSR expression due to hypercalcemia—which applies to pHPT—might be ineffective. However, in a murine model with reduced expression of CaSR in the parathyroid glands, a Japanese group could show that cinacalcet was still active in these animals despite low numbers of target receptors and already high calcium concentrations. A negative correlation between the potency of cinacalcet HCl and CaSR expression in parathyroid glands suggested that cinacalcet HCl exerts its effect via CaSR and that the potency of cinacalcet HCl depends on the degree of CaSR expression. Even in the CaSR hypoexpression state induced by severe hyperparathyroidism, the resistance to CaSR activation was overcome by increasing the dose of cinacalcet HCl [3]. 

In this paper, literature regarding cinacalcet therapy in primary HPT will be reviewed and the decision algorithms for various patient subgroups will be discussed regarding parathyroidectomy and cinacalcet or other medical treatment options. The discussion will focus on safety issues, the main complications of primary HPT (hypercalcemia, and nephrolithiasis, fibrosing osteitis, neurological complications) as well as genetic risk factors. Since persisting hyperparathyroidism after kidney transplantation resembles primary HPT in many ways, this entity will also be covered in a separate paragraph.

## 2. Parathyroidectomy

Dillon et al. reviewed the literature regarding the treatment of pHPT with a focus on cinacalcet. A MEDLINE (1965–June 2009) and bibliographic search of the English-language literature was conducted using the search terms cinacalcet, calcimimetics, primary hyperparathyroidism, and treatment. All articles identified in the search were included. 

They concluded that parathyroidectomy is curative for many patients with pHPT; however, there are further options for several patient subgroups as follows:

1. patients who are not surgical candidates or who refuse surgery,
2. those with refractory pHPT after parathyroidectomy,
3. patients who are asymptomatic and therefore unwilling to undergo surgery although they would benefit from it.

Cinacalcet may be considered to reduce serum calcium and parathyroid hormone serum levels in patients with pHPT who cannot undergo surgery or are unwilling to do so and those with refractory pHPT after parathyroidectomy. As the effects of cinacalcet on bone mineral density are uncertain, and bone biopsies are rarely available, more frequent monitoring of bone mineral density may be an option. The authors recommend future studies to evaluate the effect of cinacalcet on complications of pHPT [4]. 

In 80% of cases, pHPT is due to a single parathyroid adenoma, for which surgery is considered the treatment of choice based on guidelines established by the National Institutes of Health Consensus Panel. However, since success rates of the operation are less than 100% and vary between centers, pharmacotherapy with agents such as cinacalcet could be an alternative in a subset of patients with persistent hypercalcemia after one or more surgical interventions [5]. 

 Indications for parathyroidectomy according to the NIH consensus include a DEXA scan T score less than −2.5 standard deviations from the mean, serum calcium 1 mg/dL above the upper limit of normal, 24-hour urine calcium above 400 mg/24 h, age less than 50 years, or a creatinine clearance that is 30% below age- and sex-matched controls. However, at present many patients are identified at earlier stages of the disease. As these patients often have cognitive impairment and latent depression they would already benefit from an early parathyroidectomy in terms of quality of life as measured by the SF-36 questionnaire [6], but, because of relatively few symptoms most of them are unwilling to have surgery performed.

Presently, no data are available on bone biopsy results in pHPT patients treated with cinacalcet and no improvement in bone mineral density scores was found. But the same applies to parathyroidectomy, which also fails to achieve higher DEXA scores in many cases: In a study with post-menopausal women with pHPT the score had remained unchanged or even decreased one year after surgery in 52 out of 103 patients [7].

Obviously the risk of general anaesthesia must be considered in all operations. This risk varies highly between centers and geographical locations. Death rates from airway complications during general anaesthesia may be very low at 1 : 48200 throughout France [8], but anaesthesia-related mortality can be 100 times higher than that (1 : 482) in a district hospital in the developing world [9].

## 3. Controlling HPT Complications

### 3.1. Hypercalcemia

All studies confirm that cinacalcet effectively controls hypercalcemia while only modestly reducing PTH levels in pHPT. As Sajid-Crocket et al. point out, primary hyperparathyroidism (HPT) is the leading cause of hypercalcemia in the outpatient setting, and it is treated primarily by parathyroidectomy. They conducted a retrospective chart review from 2004 to 2006 to investigate the efficacy of cinacalcet in reducing serum total calcium ionized calcium, and parathyroid hormone (PTH) in patients with primary HPT. Patients were started on cinacalcet if they met at least one indication for parathyroidectomy. The primary outcome was normalization of serum calcium. A total of 18 patients with primary HPT were started on cinacalcet: 16 men and 2 women with a mean age of 70 years. Mean baseline serum calcium was 10.60 ± .53 mg/dL; ionized serum calcium was 1.45 ± .07 mmol/L; and serum PTH was 141 ± 78 pg/mL. After treatment with cinacalcet, the mean serum calcium decreased to 9.46 ± 3.4 mg/dL, ionized calcium decreased to 1.26 ± .06 mmol/L, and PTH decreased to 108 ± 64.5 pg/mL. Ninety-four percent of the patients on cinacalcet had normal total serum calcium, 81% had normal serum ionized calcium, whereas only 25% had a normal serum PTH level. Cinacalcet normalizes serum calcium in most patients while only modestly reducing serum PTH levels [10]. 

The group of Peacock and Shoback found successful and persistent control of hypercalcemia in two studies, including a long-term study lasting up to 5 years. [11, 12]

Cinacalcet was effective in normalizing calcium and phosphorus concentrations in patients with persistent pHPT after unsuccessful parathyroidectomy [13]. 

The treatment was well tolerated. Highest doses were reported by Marcocci et al. [14], who enrolled patients with otherwise intractable pHPT (defined as either failed or contraindicated surgery) in 23 centres in Europe, the US, and Canada. They administered up to 90mg cinacalcet 4 times per day. Although metabolized primarily in the liver, cinacalcet was successfully used in a patient with Child-Pugh B class primary biliary cirrhosis who refused to have surgery [15].

Possible medical treatment options for pHPT-induced hypercalcemia include estrogens, raloxifene, bisphosphonates, and calcitonin, apart from cinacalcet.

### 3.2. Hyperphosphaturia

Hyperphosphaturia is one of the features of hyperparathyroidism and contributes to hypophosphatemia and fibrosing osteitis. Parathormone upregulates fibroblast growth factor 23 (FGF-23) and several other so-called phosphatonins (phosphaturic peptides), which in turn inhibit Na(+)-Phos(i) transporters in renal epithelial cells and reduce phosphate reabsorption from the proximal tubuli. [16]. Cinacalcet was found to reduce hyperphosphaturia and increase serum phosphate levels into normal or almost normal ranges in all studies.

### 3.3. Fibrosing Osteitis

This complication of pHPT is the most long-term one. The duration of followup in the longest study on cinacalcet in pHPT patients conducted so far was 5 years: Peacock et al. examined long-term tolerability, safety, and efficacy of cinacalcet in pHPT patients including bone parameters in a 4.5-year open-label extension study, conducted at 14 study centers in the United States. Forty-five subjects with pHPT from a double-blind, placebo-controlled, 1-year trial were continued into this study. After the parent study, all subjects were treated with 30 mg cinacalcet twice daily, increasing to 50 mg twice daily during the 12-week titration phase if serum calcium levels were 10.3 mg/dL or higher and then maintained on cinacalcet for up to 4.5 years. Assessments included serum calcium, PTH, phosphate and alkaline phosphatase, and area bone mineral density (aBMD). Vital signs, safety chemistries and hematology, and adverse events were monitored throughout. Compared with baseline, cinacalcet treatment improved biochemical measures of pHPT including reducing serum calcium and PTH and increasing serum phosphate with slight increases in alkaline phosphatase. No changes in z-scores of aBMD at spine, hip, or wrist were seen with annual percent changes, consistent with reports for untreated postmenopausal women or pHPT patients. Safety biochemistries remained normal, and adverse events (most commonly arthralgia, myalgia, diarrhea, respiratory infection, and nausea) were mild to moderate in severity. In conclusion, treatment of pHPT patients with cinacalcet for up to 5.5 years maintained normocalcemia, reduced plasma PTH, increased serum phosphate to almost normal levels (due to reduction of hyperphosphaturia) with no significant effects on aBMD, and was well tolerated [17]. The fact that alkaline phosphatase increased over time in this study probably reflects the ongoing course of the disease, including continuous bone repair mechanisms. 

 Severe bone pain due to primary HPT has become a rare event, since the condition is currently diagnosed much earlier than it used to be. In clinical trials with secondary HPT, cinacalcet was found to be very effective for PTH-induced bone pain. Parathormone stimulates bone remodelling and FGF-23 synthesis in the bone. In conclusion a beneficial effect still has to be proven in bone biopsy studies, but the calcium controlling effect was maintained over long-term administration and parathyroidectomy fails to achieve increased BMD scores in most patients, too, especially in post-menopausal women.

### 3.4. Nephrolithiasis

At this point it is necessary to explain the mode of action of calcimimetic therapy in the renal tubules. While the calcium-sensing receptor is expressed almost ubiquitously, crucial locations in HPT therapy are the parathyroid glands, the intestine (to control calcium resorption) and the kidneys. Calcimimetics act as allosteric activators of the CaSR, enhancing its sensitivity towards calcium ions: in the renal tubules, this leads to decreased calcium reabsorption. 

 The fact that activation of the CaSR in the renal tubules triggers calciuria with subsequently elevated risk of nephrolithiasis can be neglected in a state of renal insufficiency such as secondary HPT but not in primary HPT or persisting HPT after kidney transplantation. A simultaneous decrease of PTH and serum calcium levels due to the therapy apparently balances out this tendency towards hypercalciuria in many patients as, for example, one study found that cinacalcet safely normalized serum calcium and lowered PTH concentrations without increasing urinary calcium excretion in the study subjects, indicating the potential benefit of cinacalcet as a medical treatment for primary hyperparathyroidism [10]. Several other studies did find an increased 24 h excretion of urinary calcium, while at the same time the morning fasting urine calcium/creatinine ratio was decreased, which might be attributed to the slight diuretic effect of cinacalcet in subjects with normal renal function [11]. However, the PTH decreasing effect is much less pronounced than the calcium lowering effect. 

In the long-term study conducted by Peacock et al., 2 out of 45 patients (4%) developed nephrolithiasis. Genetic data of these patients were not reported, but we do know that patients with the Arg990Gly polymorphism, which leads to permanently increased sensitivity of the CaSR, have been found to carry an increased risk of calcium stone formation [18]. Moreover, these patients respond more strongly to cinacalcet as compared to patients without the polymorphism [19, 20], so that an increased risk of nephrolithiasis due to calcimimetic therapy for pHPT cannot be ruled out especially in this patient population. Apart from cinacalcet, possible medical treatment options include estrogens, raloxifene, bisphosphonates, calcitonin.

### 3.5. Cognitive Parameters

Primary HPT impairs cognitive function even before clinical symptoms are noticed by the patient. These symptoms may resemble depression with memory deficits, mood changes and sleeping problems. In a study, early parathyroidectomy improved scoring in cognitive assessment questionnaires [5]. Similarly, cinacalcet was also able to improve health-related quality of life (HRQOL) scores, too, including cognitive tests as compared to the placebo group.

## 4. Genetic Risk Factors Interfering with Calcimimetic Therapy

The genetic background of pHPT patients does influence the decision which therapy to choose. Sporadic nonfamilial parathyroid adenomas, associated with the cyclin D1/PRAD1 oncogene [17], are the classical indication for surgery, while hypercalcemia due to hyperplasia in all parathyroid glands as in the MEN1 syndrome (associated with MEN1 tumor suppressor gene) could be treated with cinacalcet.

Regarding secondary hyperparathyroidism, there are significant differences in the progression of the disease between patients of different ethnicities: while black patients require higher vitamin D doses than non-blacks [21], Asian patients show slower progression of the disease than non-Asians with the same stages of renal insufficiency [22]. 

Even though no mutations of the calcium-sensing receptor gene have been found in parathyroid adenomas so far [17], the Arg990Gly polymorphism of this gene is important for calcimimetic pHPT therapy as a risk factor for nephrolithiasis. It is important to note that the glycine allele of this polymorphism, which renders the receptor more sensitive to calcium ions, is the most common allele (>90%) in Asians. Patients with Arg990Gly have better outcomes in pHPT [2]—it is intriguing to speculate, that the slower progression in Asian secondary HPT patients is due to their carrying the more sensitive CaSR allele.

Calcium nephrolithiasis is genetically determined [23], and since pHPT is the most common cause of this condition apart from renal tubular acidosis, it should be considered when planning the therapy.

## 5. Persisting Hyperparathyroidism after Kidney Transplantation

This condition is a clear indication for calcimimetic therapy, since untreated it results in permanent damage to the bones and parathyroidectomy is an unnecessary operative risk in these immunosuppressed patients: after one year cinacalcet can usually be discontinued [24]. Additionally, parathyroidectomy often either fails to control hypercalcemia or leads to permanent hypoparathyroidism with all related risks for graft and patient survival. Recently an improvement in bone density by cinacalcet was reported for this patient population [25]. From the clinical perspective, the disease develops from secondary HPT, but it resembles pHPT in that it leads to hypo- rather than hyperphosphatemia due to increased phosphaturia.

## 6. Conclusion

Although parathyroidectomy remains the only curative approach to the majority of pHPT cases, medical treatment with cinacalcet HCl has been proven to be a reasonable alternative for several patient subgroups. Hypercalcemia as the main clinical issue can almost always be sufficiently controlled as well as hypophosphatemia, PTH levels may be lowered, and cognitive as well as HRQOL parameters improve with the treatment. An increase in bone mineral density DEXA scan scores was not demonstrated in pHPT cinacalcet trials, the same applies to more than half of patients after parathyroidectomy. In 10–15% of patients the etiology of primary HPT is hyperplasia in all glands rather than a separate adenoma, so that medical therapy is first choice in these patients. An increasing proportion of patients are detected in lab screens showing hypercalcemia before developing symptoms. These patients should be treated early however; they are generally reluctant to undergo surgery as they are a- or oligosymptomatic. Other patients with persisting HPT after failed surgery or inoperable ones due to co-morbidities should also be offered medical therapy. 

Nephrolithiasis occurred in 4% of patients receiving cinacalcet long-term for pHPT. It was not found to occur more often in cinacalcet trial groups in several short-term studies, but urine calcium excretion as one major risk factor of this complication of primary HPT may increase with cinacalcet therapy. Patients with the Arg990Gly polymorphism of the calcium-sensing receptor, who carry a higher risk of calcium stone formation and respond more sensitively to cinacalcet, should therefore be offered alternative medical treatment options such as bisphosphonates, calcitonin, estrogen, or raloxifene.

The dosing patterns are also debatable. While most trials used twice daily dosing of cinacalcet, an increased frequency of three or four doses per day would mimic the pulsatile PTH-pattern which has been found to be beneficial for the prevention of PTH induced osteopathy [26].

In summary, the statement of Franceschini et al. [27], which they made in their early review article in 2003, has passed the test of time: cinacalcet HCl is indeed an agent not only for the management of secondary but also primary hyperparathyroidism.
